# Review of the Case of Ann Fooks, with Arguments to Prove Her Having Practised a Deception

**Published:** 1814-04

**Authors:** James Gibbon


*For the Medical and Physical Journal.*

### *Review of the Case of Ann Fooks, with Arguments to prove her having practised a Deception; by* Mr. James Gibbon.

(*Concluded from p. 209.*)

ABOUT five or six weeks after the reputed urinous vomiting had ceased, Dr. Y. accompanied me to see A. F.; we found her in the state which Mr. Pulley has described. (He and the other medical gentlemen can testify that I was strictly correct, in speaking of her apparently healthy and well-nourished state of body.) The reputed meteorismus ventriculi still existed, although the urine flowed very properly from the bladder. I pointed out to Dr. Y. the probable cause of this inflation: he denied the possibility of its being owing to the voluntary contraction of the lower abdominal muscles, muscles, because he affirmed that no person could contract them so partially as to cause such an appearance of inflation. I asserted that I could myself, that any person might, and that impostors have frequently been known to do it. Still he was unconvinced. We agreed to refer it to his medical brethren of the Infirmary, Mr. Short and Mr. Pulley; and within a day or two, they met us at the lodgings of A. F. The meteorismus ventriculi had, however, completely vanished, and Dr. Y. had forgotten that he gave a positive opinion of the impossibility of the thing. I need hardly say that Mr. Short and Mr. Pulley agreed most fully with me, as I think every physiologist must. It is easy to imitate the precise appearance which the body of A. F. presented; and which she was in the habit of showing to all descriptions of visitors, for the probable purpose of exciting compassion. I had never, before the visit above alluded to, when Dr. Y. was present, declared implicitly in *her hearing* that I was firmly persuaded the tumefaction was voluntary. It is a most strange, but undeniable fact, that the meteorismus ventriculi took wing so soon after I declared (in the hearing of A. F.) my opinion of its cause, and my conviction that any other medical man but Dr. Y. must necessarily be of my opinion. I was told that the inflation of the stomach subsided in consequence of her having bloody stools, and passing bloody urine; but here again was matter of surprise, for upon my desiring to see these discharges, I was informed they had *ceased*, and that was only *three days* after the subsidence of the inflation.

At this last meeting or consultation, when Messrs. Short and Pulley attended, Dr. Y. conversed with us all for a considerable time, upon the probabilities of there having been deception practised, in regard to the reputed urinous vomiting of A. F. He did not agree with us, of course, but parted with declaring that he would publish the case, in justification of his own opinions. This was before the "document" was signed. From this period, which was early in June 1812, I considered Dr. Y. as being voluntarily pledged to publish the case. This alarming threat was held over our heads, and we were all decidedly at issue with him.

Was it improper or indelicate then in me to ask Dr. Kerr and Dr. Alvey to give an opinion in justification, or condemnation, of my own opinion?

I paid Dr. Yeats the compliment of sending to his house to say that some medical gentlemen were going with me to see A. F. I deny that it was ungracious in me to persevere in taking them, because he was from home. Did he think it necessary to inform me when he asked a medical friend to visit A. F.? He never did. He must be aware that nothing new could be the subject of our conversation. The same facts which were fully stated to him at the former meeting, were now mentioned to Dr. K. and Dr. A., Messrs. Short, Pulley, and myself. The young woman was questioned by all the party, as to the different symptoms and declarations. The state of her general health at that moment was made the subject of particular inquiry. Her own account of the urinous vomiting, and particularly of the state of her feelings, when it was supposed to commence and to cease, were minutely attended to. Although Dr. Y. was absent, he either did know, or ought to have known, all we could possibly learn from A. F. seeing he was already pledged to publish the whole of the case. If I regretted that he was from home, does that mean he is authorised to say our meeting has an ungracious appearance? He is not authorised* to

* In his last communication he very adroitly contrives to throw all the blame upon me, but contrary to his recently-published opinions, I have a letter from him of the 27th of August, 1812, which perfectly exonerates me and them from every imputation of illiberal motives. He declares that old women and nurses only can be retailers of such declarations as he has lately indulged in respecting our meeting, when the document was signed.

repeat repeat this observation, in the manner he does, after the full explanations he has received. If he thought it was essential for him to be present with Dr. K. and Dr. A. when they saw A. F. he was at liberty to send for them the next day. They maturely weighed the question before they committed their opinion to writing: the "document" we signed *agreed with an opinion which Mr. Short and Mr. Pulley gave me in writing*, after Dr. Y. and I met them, at the time above-mentioned. I neither did, nor was I called upon to express much sorrow at the absence of Dr. Y. After he had pledged himself to publish the case, and Mr. Pulley and I to answer him, surely we were each at liberty to ask the opinion of our respective medical friends, upon the subject in dispute. Dr. Y. was, I believe, at this very time, when he complains so much of my conduct, in the habit of showing her case, as reported in his case book, to many of his friends who were interested in the business. He was likewise in the habit of showing the case of Dr. Senter, with the view of biassing the public mind in his favor. Can he then, with the shadow of justice, accuse me of acting ungraciously, for assuring the public that many medical gentlemen of respectability were of a similar opinion with me, in regard to the existence of imposture in this case? Dr. Y. labors hard to make it appear that I acted with impropriety towards him, subsequently to the existence of the "document." His endeavours must be vain, because at the moment of my presenting him with a copy of it, he told me angrily and sarcastically "that he had no doubt of being able to controvert the opinion of five such great names as he saw attached to it." In the daily expectation then of seeing a publication which was to clear up the injured innocence of A. F. and put to confusion the opinions of her adversaries, I passed over many months. I had, however, the consolation of knowing that other medical men of great experience and celebrity were to bear the brunt of the attack with me; and I must confess that I had no great fears about the result of a public discussion of this curious case.

As Dr. Y. desires us to recollect that his remarks are purely defensive, let me shortly place the question in a fair point of view. I unfortunately meet with a case of illness in which I think there are just grounds for suspecting imposture: knowing well that a large portion of the inhabitants of Bedford felt unusual interest in this case, I must have committed an act of insanity in making this opinion public, if I had not felt assured that the circumstances of imposture were clear and convincing. (As far as I know they have proved convincing to every medical mind, with one exception.)

Dr.

Dr. Y. having believed in all the assertions of A. F. which I thought incredible, pledges himself to prove their veracity. Instead of doing this, he acts in a manner which I think very unjust towards me. Is it not in the highest degree unmanly to attempt to injure the medical reputation of a young man, recently established in a professional capacity? By publishing upon ischuria, without giving the case of A. F. Dr. Y. did this. It is sophistry to deny it. I felt an honest indignation at the appearance of his first paper; which I could not but show towards him, when I sent my statement of the case of A. F. to the Journal. He was never called upon to redeem his pledge, and prove the veracity of A. F. because I deprecated a controversy.

When Ann Fooks was said to be in the state of insensibility described by Dr. Y. in page 32 of your Journal for January last, Mr. Williams and I saw her. We are of opinion that it was designedly a feigned insensibility. When I went into the room, her mother told me that she knew persons by the touch,* and that if I would sit close by the bed, and let her feel me, she would be able to *mutter out* who I was, either immediately or soon after I was gone. A person of great respectability in the town can testify to a similar occurrence, whilst visiting her during the supposed state of carus ischuriosus. Would the celebrated men whose names Dr. Y. mentions (page 117), have thought these tricks compatible with a state of *real coma?* †

Well might I suppose that Ann Fooks was under the sole care of the druggist, because he occasionally visited her during this long period, which passes almost unnoticed in the history of her case: he was to report to Dr. Y. what medicines he thought fit to venture upon; and, although Mr. Williams and I were repeatedly desired to prescribe, we thought it wrong to do so, for reasons before stated. At this time, whilst A. F. was supposed to be suffering under "carus," her mother assured Mr. Williams and me, that she thought the urine was working about her, and would soon return to its natural channel.

JAMES GIBBON.

---

* Upon asking how she (the mother) could tell this, she informed me that when her daughter came a *little to herself*, she could tell what had passed, and who had been there, whilst she lay in the fits.

† Dr. Y. has also omitted to inform you that A. F, in one part of her illness, had fits which continued for many hours, during which she barked exactly like a dog. The druggist, Mr. Palgrave, exhibited *a considerable portion of her lungs in spirits,* which she declared she coughed up.

